# Perspectives of Caregivers of Kidney Transplant Recipients and Transplant Candidates About Kidneys From Donors With Hepatitis C Virus Infection

**DOI:** 10.1016/j.xkme.2026.101410

**Published:** 2026-05-13

**Authors:** Matthew D. Kearney, Caroline S. O’Brien, Sabrina A. Emms, Eugenia I. Manrique, Barbara Gonzalez Alarcon, Ella N. Pokrifka, Adam Mussell, David S. Goldberg, Peter P. Reese

**Affiliations:** 1 Mixed Methods Research Lab, Department of Family Medicine and Community Health, University of Pennsylvania Perelman School of Medicine, Philadelphia, PA; 2 Leonard Davis Institute of Health Economics, University of Pennsylvania, Philadelphia, PA; 3 School of Nursing, University of Pennsylvania, Philadelphia, PA; 4 Department of Medicine, Division of Digestive Health and Liver Diseases, University of Miami Miller School of Medicine, Miami, FL; 5 Center for Clinical Epidemiology and Biostatistics, University of Pennsylvania Perelman School of Medicine, Philadelphia, PA; 6 Vanderbilt Center for Transplant Science, Vanderbilt University Medical Center, Nashville, TN; 7 Section of Surgical Sciences, Vanderbilt University Medical Center, Nashville, TN

**Keywords:** Kidney transplantation, qualitative research, caregivers, hepatitis C virus

## Abstract

**Rationale & Objective:**

Kidneys from deceased donors with active hepatitis C virus infection (HCV-RNA+) are commonly transplanted into HCV-negative recipients, yet little is known about the perspectives of their caregivers. This study’s objective was to better understand caregiver experiences about the transplantation of HCV-RNA+ donor organs, with particular attention to caregiver educational needs and perception of risks.

**Study Design:**

Cross-sectional study at 2 centers.

**Setting & Participants:**

We conducted a qualitative study of 20 adult caregivers of patients in the THINKER-NEXT trial, in which patients consented to receive kidney transplant offers from HCV-RNA+ donors. At the time of the interview, participants were either providing support to waitlisted patients or to recipients of HCV-RNA+ donor kidneys.

**Analytical Approach:**

Semistructured interviews explored caregiver knowledge, concerns, and experiences related to HCV infection and transplantation. Transcripts were coded thematically using an iterative, consensus-based approach.

**Results:**

Caregivers described 4 central themes: 1) patient and caregiver dynamics shaped transplant decisions and caregiving responsibilities; 2) knowledge and attitudes about HCV influenced acceptability of organs with HCV, with initial concerns often offset by the desire to avoid dialysis; 3) privacy, stigma, and the strength of social support networks influenced willingness to disclose transplant details; and 4) cautious optimism and uncertainty in preparing for life after transplant. Many caregivers expressed a need for clearer guidance on HCV risk, caregiver safety, and posttransplant care.

**Limitations:**

Transferability may be limited because participants were caregivers of clinical trial participants at academic medical centers.

**Conclusions:**

Caregivers were supportive of transplantation from donors with HCV infection. However, they identified gaps in education and posttransplant support. Integrating caregiver perspectives into education, particularly about infectious risk mitigation and caregiver safety, may better enable patients and caregivers to thrive after transplant. These insights are particularly relevant as practices evolve toward transplanting organs with diverse infectious risks, including xeno-organs.

## Plain-Language Summary

Some kidney transplants come from donors with infections such as hepatitis C virus (HCV) infections, but little is known about the experiences of caregivers of patients considering these organs. This study explored the experiences of 20 caregivers who had received kidney transplants or were on the waiting list. All the patients had received kidney transplants from donors with active HCV infection or had agreed to consider offers of kidneys from donors with HCV. Caregivers generally supported the practice of transplanting kidneys from donors with the HCV infection because of a desire to improve their patients’ quality of life. However, many caregivers expressed a need for clearer guidance on the risks of HCV infection, caregiver safety, and posttransplant care.

## Introduction

Kidney transplantation improves survival and quality of life compared with chronic dialysis,^1^^,^^2^ but the ability to access these benefits depends on the ability of patients and their caregivers to successfully navigate challenges that begin with wait-listing and continue through the life of the transplant. The need for caregiver support is so fundamental to transplantation that demonstration of adequate social support is a relative or absolute requirement in the transplant evaluation process at most centers.^3^ Yet, many knowledge gaps exist about how meeting caregiver needs might facilitate better transplant outcomes.^4^ In the setting of transplanting kidneys from deceased donors with infections such as hepatitis C virus (HCV), the experiences and beliefs of caregivers could affect the patient’s decision to accept an organ offer and expectations about posttransplant outcomes.^5^

For patients and caregivers, a common motivation for transplantation is to find relief from the burdens that dialysis places on the family.^4^^,^^6^ For patients seeking a kidney transplant (KT), the caregiver role commonly encompasses supporting the patient through the burdens of chronic dialysis while simultaneously meeting the center’s requirements to stay active on the waiting list.^4^ Caregiver duties may include assistance with transportation, supporting adherence to complex medication regimens, completion of medical testing, and helping with nuanced decision-making, such as what types of organs to accept. In addition, caregivers who reside with the patient may carry out a large share of household responsibilities and/or manage greater financial burdens.^4^^,^^7^^,^^8^ For caregivers of patients on dialysis, a meta-analysis showed they have worse quality of life than demographically matched peers and experience significant burdens as assessed using the Zarit Caregiver Burden scale,^9^ while caregivers in a qualitative study reported that their stress levels increased and their social lives and sexual relationships worsened after their partners began dialysis.^10^ While caregivers and patients may feel optimism about the opportunity of transplantation, they also typically endure anxiety about long waiting times and the potential that health deterioration related to end-stage kidney disease may render the patient ineligible for transplant.^7^

The ability to obtain a transplant often depends on the patient’s willingness to consider organs with risk factors for complications.^11^ In 2023, HCV-RNA+ deceased donors provided 5% of all deceased donor kidneys,^12^ with higher percentages in some regions. A large proportion of deceased donors carry viral infections such as cytomegalovirus, Epstein-Barr virus, hepatitis B, as well as other infections that require prophylaxis or treatment of the organ recipient.^13^ Some disease transmissions, such as cytomegalovirus, are expected, while others only become apparent after transplantation.^14^

Transplant risks bring additional implications for caregivers. First, caregivers may be asked by patients to participate in decision-making about which organs to accept, but often feel overwhelmed by the complexity of the process.^4^^,^^6^ Many donor-derived infections may be unfamiliar.^15^ In the case of HCV, caregivers could be exposed to infection if they handle sharps used by the patient and may be advised to take precautions, including barrier protection during sex.^16^ More than a hypothetical concern, potential HCV exposures via needlesticks among close contacts of recipients of organs from HCV-RNA+ donors have been reported.^17^ In a qualitative study of recipients of HCV-RNA+ donor transplants, the effect of HCV infection on relationships emerged as an important theme, with 1 patient noting that “my wife was very scared,” while conversely, other participants felt that their transplant had the effect of making relationships closer.^15^ In another study of 44 patients who were deciding whether to receive HCV-RNA+ donor organs, participants noted that advice from family and friends was very important to their decision.^5^ However, to our knowledge, no published studies have directly assessed the experiences of caregivers for patients considering or having received a HCV-RNA+ donor kidney.

The assessment of caregiver perspectives on donor-derived infection may gain increased salience as the transplant field gains experience with genetically engineered porcine organs, where risks of animal-to-human infection transmission to recipients and close contacts remain a key concern.^18^^,^^19^ For the present study, we aimed to assess the experiences and needs of caregivers of patients wait-listed for kidney transplantation who had opted in for offers of kidneys from HCV-RNA+ donors and recipients of these KTs.

## Methods

### Study design, setting, and participants

We conducted an observational qualitative study using semistructured interviews with caregivers of adult patients with ESKD. The patients were enrolled in the single-arm, open-label THINKER-NEXT clinical trial (NCT 04075916) at the Hospital of the University of Pennsylvania or the University of Miami/Jackson Memorial Hospital/Miami Transplant Institute sites, who were as follows: 1) candidates for KT on the waiting list who had consented to receive offers of kidneys from HCV-RNA+ deceased donors, or 2) recipients of KTs from HCV-RNA+ donors. We refer to study caregivers in the first group as “pretransplant caregivers” and in the second group as “recipient caregivers.” The trial involved counseling patients about donor-derived HCV infection and provided antiviral therapy post-transplantation for recipients of HCV-RNA+ donor kidneys. Caregiver participants were identified by speaking to THINKER-NEXT clinical trial patients and requesting permission to contact their caregivers (institutional review board protocol #852630). Item S1 further describes the THINKER-NEXT clinical trial.

### Data collection and analysis

Interviews lasted between 10 and 20 minutes, were conducted via phone by OBC and SAE, and were scheduled at the participant’s convenience. With participants’ consent, interviews were audio recorded and transcribed verbatim, and transcripts were subsequently reviewed to remove identifying information. Deidentified transcripts were qualitatively coded and analyzed to identify emergent themes related to patient and caregiver decision-making about receiving transplants from HCV-RNA+ donors. During interviews, caregivers were asked about their relationship with their patient, understanding of the trial and HCV, and what concerns they had about an organ transplant with HCV. Caregivers of KT recipients from HCV-RNA+ donors were additionally asked about posttransplant experiences, counseling, and communication about HCV. After completing 20 interviews, the study team determined that thematic saturation had been achieved and additional interviews would not be necessary.^20^^,^^21^ Items S2 and S3 provide the questions addressed to caregivers.

Investigators developed a codebook using a grounded theory-based approach, including an open coding process to iteratively identify themes and patterns present in the data.^22^, ^23^, ^24^ OBC and SAE were principally responsible for codebook development, with all authors providing feedback before coding began. Codes included both emergent and a priori topics, including knowledge about HCV infection, the transplant process, caregiver and patient characteristics, and relationships between both caregivers and patients, as well as between caregivers and care providers. We included topic codes for mentions of dialysis and the transplant waitlist.

Two researchers (OBC and SAE) co-coded 5 transcripts (∼20% of the total sample) and met to resolve discrepancies in coding and adjusted the codebook in NVivo Qualitative Data Analysis Software (QSR International, version 14). After completing the interrater reliability process, the remaining transcripts were coded by OBC (final average κ = 0.97). The study adhered to the COnsolidated criteria for REporting Qualitative research (COREQ) guidelines.

## Results

### Sample characteristics

A total of 20 caregivers participated out of 33 initially contacted by the study team. Nine participants were caregivers for patients who had received KTs from HCV-RNA+ donors, while the remaining 11 were caregivers for waitlisted candidates. Table 1 shows participant characteristics. The mean age was 46.7 years; 70% were female, and 40% identified as Black.

**Table 1:** Characteristics of Caregivers of Recipients of Kidney Transplants From Donors With Hepatitic C Virus (HCV) Infection or Waitlisted Patients Eligible for Kidney Offers From Donors With HCV Infection

Characteristic	Caregiver (N = 20)
Age in y (± SD)	46.7 (15.2)
Female, n (%)	14 (70)
Race, n (%)	
White	12 (60)
Black	8 (40)
Neither White nor Black	0 (0)
Patient transplant status/Patient-caregiver Relationship	
Caregiver of waitlisted patient, n (%)	11 (55)
Spouse	4 (20)
Parent	2 (10)
Other relative (eg, cousin)	2 (10)
Other nonrelative (eg, friend)	1 (5)
Grandparent	1 (5)
Sibling	1 (5)
Caregiver of kidney transplant recipient, n (%)	9 (45)
Spouse	7 (35)
Parent	1 (5)
Other nonrelative (eg, friend)	1 (5)
Patient-caregiver relationship, n (%)	
Spouse or partner	11 (55)
Sibling	1 (5)
Child	3 (15)
Other	5 (25)

### Caregiver interview themes

**Figure 1:**
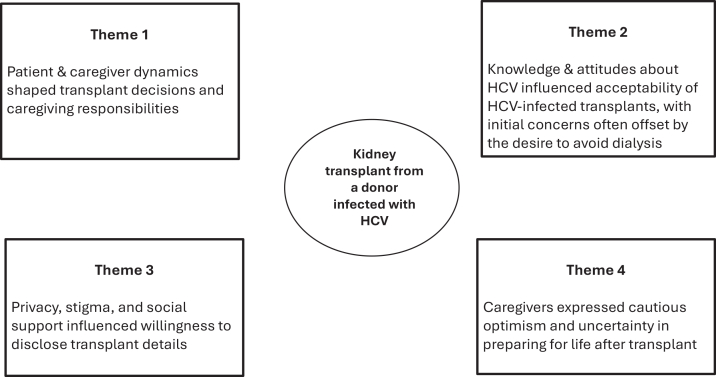
Four themes are elicited from caregivers of patients with kidney failure about transplantation with kidneys from donors with hepatitis C (HCV) infection.

There were no clear differences between the 2 groups in motivations to join the trial, understanding of potential risks and benefits, or desired outcomes from a KT. Figure 1 shows the primary categories of themes that emerged from qualitative analysis: 1) factors influencing transplant decision-making; 2) factors influencing trial acceptability; 3) desires for privacy in decision-making; and 4) future planning and considerations. We described each theme below, supported by exemplar quotes. When indicated, themes were elaborated via subcategories.

#### Theme 1: Patient and caregiver dynamics shaped transplant decisions and caregiving responsibilities

##### Subtheme 1a: caregiver and patient relationship

When asked to describe the people they cared for, caregivers generally reported patients had been navigating both kidney disease and its treatment for several years. Caregivers frequently discussed the toll of being on transplant waitlists, as well as how harsh dialysis had been for their patients (Table 2). As discussed further, time on dialysis was frequently mentioned as a motivation for joining the THINKER-NEXT clinical trial.

**Table 2:** Themes and Subthemes From Interviews With Caregivers

Theme 1: Patient and caregiver dynamics shaped transplant decisions and caregiving responsibilities
Subtheme 1a: caregiver and patient relationship
“Well, they told me [a transplant] would allow him to have more free time. He wouldn't have to be on dialysis anymore, it would give him a better quality of life back.” (Recipient Caregiver 100-041)
“As I said, I was hesitant in the beginning because I knew it was hepatitis A, hepatitis B, hepatitis C. And so that's when I started reading up on it, and he explained more that he gave me a pamphlet, I think that they sent him. I was down with it with him.” (Pretransplant Caregiver 100-063)
Subtheme 1b: Caregiver as medical provider
“I had moments, last week I had a breakdown. But he didn't know, so I called my best friend. I just needed to let it out to talk to somebody, because it's like I'm not a nurse. I have to do everything for him, and it's hard . . . And when he was in the hospital, he didn't want the nurses to clean him or wipe his butt . . . So I had to actually take the role of the nurse in the hospital, because he didn't want anybody to do anything for him. So that was hard. It was overwhelming. Having a 7-year-old, a house, and going to work.” (Recipient Caregiver 800-058)
“We do have family discussions, but I am in health care, so most of the time I do have the main conversations with him and then, of course, with the larger family.” (Pretransplant Caregiver 100-069)
“And my son too, he said [this trial] is a good idea because we asked him, he's a resident too in the [Medical School], and he knows about the -- we asked him, and he told him, ‘yes, go ahead. Do it. It's ok. No problem.’” (Pretransplant Caregiver 100-100)
Theme 2: Knowledge and attitudes about HCV influenced the acceptability of HCV-RNA+ transplants, with initial concerns often offset by the desire to avoid dialysis.
Subtheme 2a: Impact of transplant waitlist
“[Being on the waitlist] can be long and grueling, and I think it can be depressing at times. I think it's monotonous. They do the same thing every day, and it's wearing. It definitely is wearing. You're just waiting, like waiting for something. So, I don't know. That's all I can say; it just wears on you. It's too bad that it's going to take forever . . . You put a lot of stuff on hold waiting for things to be easier.” (Pretransplant Caregiver 100-048)
“There is another piece of this equation as well that I have to tell you. That's January of 2022, we received about four, possibly five, but let's just go with four calls for kidney transplants. And every one of them, there was either some type of issue with the donor. And obviously, what happens in those situations is that your emotions start taking over, but you get very excited. And then, at the last minute, you're packing, you're packing, you're ready to go, and you get a phone call, and unfortunately, this reason and that reason, that reason. So, we went through that. And I guess, as everybody else does, but we're no different, and talking to our primary care doctor, tried to explain to us, but that's not uncommon, and it can happen, it is not uncommon for that to happen six, seven times.” (Recipient Caregiver 100-024)
Subtheme 2b: Dialysis’s influence on desire for a transplant
“Well, I'll be honest with you, [Patient] really can't do much. All he does is go to dialysis, and by the time he comes home, he's tired. So, he rests all day after dialysis, and the next day, he might try to attempt to do a simple thing, and then he's tired, and he sleeps a lot. So, he can't really do much. So, even going to the stores, if I say, okay, well, let's walk around, let's say, a store that's so close. If I take him, we're in the store maybe five minutes, and he'll ask me for the car keys so he can just sit down. So, it's very exhausting for him. So, he can't even go shopping like that.” (Pre-Transplant Caregiver 100-045)
Subtheme 2c: Additional medical complexity
“I don't know, I mean, one has to be careful with -- having had the transplant anyway. So, to be honest, other than being sure that you take the medications the way that you're supposed to, I don't know that anything else is different just because of that, because I'm being careful with everything else concerning him anyway. So, it didn't seem to add an additional level of care that I recognize.” (Recipient Caregiver 100-052)
“Hepatitis C, I mean would you want to add to your list of issues? [. . .] I mean, do you want to add to what you've got going? Is it going to be better or worse?” (Pretransplant Caregiver 100-048)
“I would just say more safety precautions when it came to helping her. She needed help going to the bathroom, and I double-gloved. And when I needed to give her the shot, I was a lot more careful as well with the needle stick. I'd say that's all I did differently, I'd just give everything a little more thought than before.” (Recipient Caregiver 800-076)
Subtheme 2d: Caregiver HCV risk considerations
“But as a married woman, I don't feel that they explained to me what the exposure is. I don't want to say that he didn't care, but I think he was so distracted by the kidney that those preventative measures and make sure you do these things, I don't think that they were really, I don't know, I want to say toward me, not toward him, because he was well informed, well educated, and everything. I'm a nurse, but even if I wasn't? What was my exposure risk? [. . .] I wasn't included in that part.” (Recipient Caregiver 800-087)
“Sexually, well, because we can't have any intercourse or stuff. Can't share any food. I usually like to eat from his plate, because he is a chef, and he cooks so often, so I can't eat anything from his plate. So, we're very cautious. He's very cautious to make sure that I'm not affected.” (Recipient Caregiver 800-058)
“Well, affected in, you know, the process in the first month because I can't have contact with my wife, even kids, or things like that. And every time we eat together, and I remember times you see, okay, I don't want too much meat, you can take it. And I said, " Oh, no, I need to remember that. I can't. So, things like that. So, the same thing with cleaning everything before and after we go to the bathroom, to bed, the first month I sleep in a different room. We are in an apartment, and we have one bedroom. So, I sleep in the [living room], but this is a process that we discussed before.” (Recipient Caregiver 800-115)
Theme 3: Privacy, stigma, and the strength of social support networks influenced caregivers’ willingness to disclose transplant details.
Subtheme 3a: Stigma toward organ donors
“I think they had mentioned that they test all of their organs. So, even if it's somebody that did have a drug history, they would know, like, make sure that there's no HIV or anything. And you could also ask, ‘what is the donor's medical history or drug history,’ any kind of that behavioral stuff.” (Pretransplant Caregiver 800-185)
“I mean, I guess I just thought it made sense, because [overdose deaths are] such a huge public health problem right now. I didn't think it would be like -- I assumed that if this was a program, it wouldn't affect my dad's health at all, you know what I mean? Like that, they know something about how to like do the transplant so that it wouldn't affect him.” (Pretransplant Caregiver 100-051)
Subtheme 3b: Stigma toward organ recipients
“I don't think you will because I don't think I will share it with anybody. That's me personally. As a matter of fact, I'm very private, so I don't really talk to a lot of people. So, I wouldn't even mention it, I don't think. Only my immediate family.” (Prettransplant Caregiver 100-045)
“We discussed it with our family and friends, you know, you just ask for input and advice from other people [. . .] Just to get their opinion and what they thought of stuff.” (Recipient Caregiver 100-041)
Theme 4: Cautious optimism and uncertainty in preparing for life after transplant.
“I'm not really sure. I mean, I'm sure in the beginning it's going to be difficult cause I'm not -- well, I'm -- I guess, I don't really know. But I know, like recovery from surgery is something you have to be thinking about, and I know that there's, like with transplants generally, there's like a quant- a long quarantine period, and like I know he's gonna be on immunosuppressants and stuff. I don't really know anything specific, I guess, about how a [Hepatitis C virus] kidney would change that process.” (Pretransplant Caregiver 100-051)
“Right now we have, we can't travel, every night we're like, we have to be home at a certain time because of dialysis. So, the number one issue would be more freedom in that regard because he's always been an avid traveler and someone who likes to go away. The last year and a half, we've been pretty much just stuck in the house. So, I have to say freedom, freedom-wise, he'll be much happier. As far as our daily routine, I think we'll just switch gears from what we do now for the dialysis to just in case any issues arise because you're having to deal with the hepatitis.” (Pre-Transplant Caregiver 800-114)
“Yes, we, we truly, you know, hope and pray, you know, that [Patient] can, you know, receive a good kidney, you know, sooner than later . . . Really, that's really what we pray for every single day, you know? Because we don't know what tomorrow holds and, you know, we really want him to be in his back, you know, get his health back and have a normal functioning life. I mean, of course, it's not gonna be optimal, but at least he's going to get some of what we hope that he had back the time that he spends with his family and working and doing other things, right?” (Pretransplant Caregiver 100-069)

HCV, hepatitis C virus.

Caregivers typically mentioned how they were connected to the patient without commenting on the quality of the relationship. Caregivers tended to speak about caring and trusting relationships with shared decision-making about the patient’s health. Many caregivers said their patient talked to them about the THINKER-NEXT clinical trial, but the final decision to enroll was made by the patient themselves. While some caregivers reported initially not wanting their patient to consider HCV-RNA+ donor organs, no caregiver mentioned ongoing misgivings about the trial or the treatment plan.

##### Subtheme 1b: caregiver as medical provider

Caregivers also spoke about acting as both medical and linguistic translators between their patients and health care providers. Few caregivers mentioned disliking or feeling strained by that aspect of care work. However, some caregivers did mention struggling with caregiving if they were not trained medical providers, if they had to balance caretaking with other responsibilities (such as raising children), or if their patient asked them to caretake in place of trained medical providers (Table 2).

A substantial portion of caregivers had some medical training themselves (several were nurses or were in nursing school). Caregivers who were not themselves medical professionals often mentioned asking for advice from people close to them who did have medical backgrounds, such as grown children or extended family members.

#### Theme 2: knowledge and attitudes about HCV influenced the acceptability of HCV-RNA+ transplants, with initial concerns often offset by the desire to avoid dialysis

##### Subtheme 2a: impact of transplant waitlist

The majority of caregivers felt well informed about the risks of their patient’s participation in the THINKER-NEXT clinical trial, though when asked what they knew about transplant with HCV-RNA+ organs, they tended to focus on the benefits. Reducing time on the transplant waitlist and wanting to stop dialysis seemed to be the primary motivations for people to enroll in the THINKER-NEXT clinical study. Caregivers described how heartbreaking it was to think there was finally a kidney available to them, only to have it not be the match that their care team thought it would be, and they hoped that entering the THINKER-NEXT clinical trial would stop that kind of event from happening again (Table 2).

##### Subtheme 2b: dialysis’s influence on desire for a transplant

Caregivers and patients were strongly motivated to join the trial (and thereby get a transplant more quickly) as a way to stop dialysis, which many people described as a harsh treatment that did not give patients a very good quality of life. Caregivers described patients being too tired to go about their daily life after dialysis treatments and unable to take even short trips (Table 2).

##### Subtheme 2c: additional medical complexity

Caregivers seemed aware of what care would be like for patients after transplant, generally assuming their patients would need a similar level of care immediately after the surgery as they did before the transplant. At least 1 caregiver regarded the hepatitis treatment as “just one more thing.” However, other caregivers thought of treating HCV infection as yet another thing (Table 2).

As mentioned previously, several caregivers reported being unsure if their patient should enroll in the THINKER-NEXT clinical trial, specifically because of concerns about getting an organ with HCV infection. The majority of caregivers, even those who were medical providers, said they knew little about HCV infection and its treatment before learning about the study. In general, caregivers who lived with their patients tended to be more concerned about HCV infection and taking precautions to not get sick themselves. Those caregivers talked about being more diligent in cleaning shared spaces or taking extra precautions when providing medical support, like insulin injections.

##### Subtheme 2d: caregiver HCV infection risk considerations

One caregiver, however, felt her safety had not been adequately addressed during the transplant discussions, specifically the risk of her contracting HCV infection from her husband sexually. That caregiver commented that she knew to follow up with her husband’s care team because of her background as a medical provider, but the care team should have been more proactive in discussing how she could mitigate risk. She also recounted disagreements between her and her husband about what activities would put her at risk.

Caregivers were not specifically probed for precautions they were taking to prevent transmission of HCV infection, but those measures frequently came up as caregivers discussed their lives posttransplant. While some caregivers reported standard sanitation practices for an immunocompromised person, others mentioned precautions that indicated that they had not been well educated about how HCV infection can be transmitted; specifically, some caregivers reported limiting behaviors like kissing and not sharing plates or beds with their patient.

#### Theme 3: privacy, stigma, and the strength of social support networks influenced caregivers’ willingness to disclose transplant details

##### Subtheme 3a: stigma toward organ donors

No caregiver expressed any stigmatizing views of people who die from an overdose, focusing instead on the reduced wait time for a KT, though some caregivers mentioned they did not realize that donor injection drug use was a consideration until the transplant. The majority said they would want confirmation that the kidney still functioned and would not cause any additional complications, such as HIV infection.

##### Subtheme 3b: stigma toward organ recipients

Some caregivers mentioned that they would not share with friends and family that their loved one received a transplant with an organ with an HCV infection. However, it was unclear if that reluctance was because they were concerned about stigma or if they simply preferred to keep medical information private. An equal number of caregivers reported they would be comfortable sharing that information with friends and family or that they did include them in decision-making.

#### Theme 4: cautious optimism and uncertainty in preparing for life after transplant

Some caregivers were more focused on the immediate aftermath of surgery, when their patients would be very medically vulnerable and would need a lot of help caring for themselves; they voiced plans for sanitation protocols or about fears patients had while recovering.

Caregivers frequently mentioned being able to travel or return to work as the ideal posttransplant outcome. Others were thinking more long-term and talked about times when their loved one no longer needed to be on dialysis and would have an improved quality of life. Some caregivers said they wanted their loved one to be healthy enough to go shopping with them. Ultimately, caregivers seemed to think of a successful transplant as the end of the holding pattern of dialysis and the emotional strain of the waiting list.

## Discussion

Our study provided novel insights into the perspectives of caregivers supporting patients receiving or considering KTs from donors with HCV infection. Our findings highlighted the essential, underrecognized role that caregivers play in navigating these decisions and managing posttransplant care, complementing prior empirical research on clinical safety and efficacy of such transplants.^25^ The study advances the field by addressing caregiver views on donor-derived viral infection and the possibility of their own vulnerability. Across both pretransplant and posttransplant contexts, the caregivers described significant emotional, informational, and logistical responsibilities, often shaped by their relationship to the patient and their medical knowledge. Despite initial concerns, caregivers generally expressed strong support for participation in the THINKER-NEXT clinical trial. Many were motivated by a common desire to reduce dialysis burden and improve patient quality of life. As programs increasingly consider the use of organs with perceived or actual risk from donor-derived infections, these findings underscore the importance of integrating caregiver perspectives into transplant decision-making, risk communication, and long-term support strategies to address caregivers’ educational needs.^4^

First, caregivers acted as both emotional and medical support for patients navigating the uncertainty of HCV-RNA+ organ offers. Many were surprised at the extent to which they needed to help interpret clinical information for the patients. They also facilitated communication with providers and provided logistical and psychosocial support, usually without formal training. Transplant programs should consider developing tailored support strategies that acknowledge and address the emotional and practical challenges caregivers face, especially for those balancing multiple caregiving, work, and family responsibilities and those with limited medical knowledge.^4^^,^^8^ Our findings support growing calls to better integrate caregivers into structured shared decision-making processes in usual transplant care.^26^ While caregivers in our study were broadly supportive of accepting kidneys from donors with HCV infection, many described insufficient information about HCV infection transmission, their own safety, and posttransplant expectations. Persistent caregiver knowledge gaps indicate a need for improved education and risk communication. For example, framing caregiver involvement within a shared decision-making approach, such as the “3-talk” method, could support both patient autonomy and caregiver preparedness.^26^ Additionally, consistent with a recent systematic review of CKD research,^27^ caregivers expressed a desire to be more actively involved not just in care but also in the development of education tools and even clinical guidance. Notably, the American Society of Transplantation created the Organ Transplant Caregiver Toolkit to help caregivers better understand the transplant process.^28^ This toolkit or other resources should be expanded to address risks related to transmission of HCV and other infections.

Second, caregivers supported their patients’ decisions to consider KTs with HCV because of their potential to shorten wait times for transplants. However, caregiver understanding of HCV infection and its implications varied, and some initially expressed concerns about the associated risks. When paired with direct-acting antivirals, using HCV-RNA+ donor organs in HCV-negative recipients can be safe and effective, with cure rates exceeding 95% and some evidence of decreased waitlist mortality.^29^, ^30^, ^31^, ^32^ These outcomes validate the benefits caregivers cited, including faster transplant timelines and improved patient quality of life. Educational initiatives should be expanded to improve caregivers’ understanding of HCV infection, addressing both the medical implications and the safety measures. Continuous dissemination of up-to-date information about HCV infection treatment advances is critical to maintaining caregiver confidence throughout the transplant process. These insights hold relevance for other emerging areas in transplantation. For example, in xenotransplantation, caregivers could be exposed to zoonotic infections from the organ or even quarantined in the setting of possible donor-derived infection. To address caregiver needs in the setting of donor-derived infection risk, transplant policy organizations and professional societies should consider developing standards about the necessary information that caregivers should receive about infectious risks to patients and to close contacts.^18^^,^^19^^,^^33^

Third, while most caregivers did not stigmatize receiving an organ from a donor who died from a drug overdose, they varied in their willingness to share details about the transplant with others, reflecting differing levels of concern about privacy and perceived stigma. Health care teams should address these concerns, providing guidance on how to navigate discussions with family and community members. Broader public health campaigns might be able to reduce stigma associated with HCV infection and injection drug use, thereby promoting the acceptability of transplants from donors who commonly have histories of substance use disorder. Encouraging caregivers to engage their social support networks in the decision-making process could improve the overall caregiving experience.

Finally, caregivers were generally cautiously optimistic about life after transplant, focusing on both immediate recovery challenges and the long-term improvements in quality of life that they hoped would come with the cessation of dialysis. Clinicians should support caregivers and patients in setting realistic long-term goals, such as returning to work or resuming travel. Communicating clear, individualized guidelines for posttransplant care can help caregivers manage their roles more effectively, particularly in relation to HCV infection-specific precautions and general postsurgery care.

We acknowledge limitations, such as limited transferability. All participants were caregivers of patients enrolled in the THINKER-NEXT clinical trial. Trial participation included structured education and support for participants, potentially giving these caregivers more preparedness around the transplant experience compared with caregivers of patients receiving usual care. Next, participants were recruited from 2 US transplant centers. Regional and institutional factors may shape caregiver perspectives. For example, some Miami patients had agreed to a protocol for accepting HCV-RNA+ organs before the THINKER-NEXT clinical trial enrollment, which may have introduced selection bias. Interviews were conducted exclusively in English. As a result, cultural, socioeconomic, and linguistic diversity were limited. We also did not conduct member checking, a recognized strategy for validating qualitative analytic interpretations. Last, while caregivers discussed a range of psychosocial and logistical concerns, our study did not address issues such as insurance or out-of-pocket treatment costs, which may affect caregiver burden in nontrial settings.

In conclusion, caregivers described their multifaceted roles in supporting the medical, emotional, and social needs of their patients as they pursued transplants from HCV-RNA+ donors. Caregivers were generally supportive of transplant innovation— when provided with clear, consistent information and a pathway to address their concerns. However, caregivers identified gaps in education about posttransplant support, specifically related to infectious risk management and caregiver safety. Future initiatives should aim to systematically include caregivers in transplant education and planning, recognizing caregivers both as essential supports for patients and as individuals with distinct informational and emotional needs. Progress will likely emerge through center- and provider-based initiatives around counseling patients and caregivers, particularly among early adopters of novel practices such as transplanting organs from donors with an HCV infection. These insights may be especially valuable as the field prepares for innovations such as xenotransplantation, which may impose complex burdens on caregivers in terms of their own safety and the challenges of caring for their patients.

## Supplementary Materials

Supplementary File (PDF) Items S1

Supplementary File (PDF) Items S2

Supplementary File (PDF) Items S3
