# Supplementary material for: Perspectives of Caregivers of Kidney Transplant Recipients and Transplant Candidates About Kidneys From Donors With Hepatitis C Virus Infection
Source: Kidney Med. 2026 May 13;8(7):101410. doi: 10.1016/j.xkme.2026.101410 (PMC13312101; doi:10.1016/j.xkme.2026.101410)
Supplement: Supplementary File (PDF) — Items S1 [file mmc1.pdf]

Item S1

Overview of the THINKER-NEXT trial

THINKER-NEXT (**T**ransplanting **H**epatitis C Kidneys **I**nto **N**egative **K**idn**E**y **R**ecipients) is a multicenter, single-arm, open label clinical trial (NCT 04075916). Two sites included the Hospital of the University of Pennsylvania and the University of Miami/Jackson Memorial Hospital/Miami Transplant Institute.” The trial enrolled adult ($\geq$18 years) kidney transplant candidates without hepatitis C virus (HCV) infection on the transplant waiting list who consented to kidney transplantation from a deceased donor infected with HCV, followed by treatment with a direct acting antiviral. The primary outcome is post-treatment sustained virologic response (SVR) to direct-acting antiviral (DAA) [Time Frame: Baseline to 24 weeks]. The one-year allograft function and one-year risk of CMV infection will be compared between THINKER-NEXT kidney transplant recipients and matched recipients who received hepatitis C uninfected kidney transplants (these patients are called Transplant Cohort). The survival rate of patients opting-in for offers of kidneys from HCV-viremic donors will be compared to the survival rate of matched comparators from the kidney transplant waitlist who did not consent to receive offers of a HCV-viremic kidney. Lastly, renal pathologic findings will be compared among HCV-viremic donors and HCV-negative comparator donors.

The trial involved counseling patients about donor-derived HCV infection and provided antiviral therapy post-transplantation for recipients of HCV-RNA+ donor kidneys. Participants consented to follow-up while on the waiting list and, if transplanted with a

kidney from an HCV-RNA+ donor, patients consented to study visits up to one year post-transplantation. The trial was funded by the National Institutes of Health. Study drug to treat HCV infection was donated by Gilead. Dr. David Goldberg and Dr. Peter Reese were multiple principal investigators of the THINKER-NEXT trial.

Caregiver participants for this qualitative study were identified by speaking to THINKER-NEXT patients and requesting permission to contact their caregivers (IRB protocol #852630).
