# Supplementary material for: Perspectives of Caregivers of Kidney Transplant Recipients and Transplant Candidates About Kidneys From Donors With Hepatitis C Virus Infection
Source: Kidney Med. 2026 May 13;8(7):101410. doi: 10.1016/j.xkme.2026.101410 (PMC13312101; doi:10.1016/j.xkme.2026.101410)
Supplement: Supplementary File (PDF) — Items S2 [file mmc2.pdf]

Item S2: Interview guide

Caregivers of people on the transplant waitlist

Hello. My name is _______________ and I work with the doctors on the transplant team at Penn/Miami. We are interested in learning about what it is like to be a family member or caregiver of someone who is waiting for a transplant. We want to learn about this so that we can help patients and families during this process. Since you are a family member or caregiver of someone waiting for a transplanted organ, we consider you an expert in this. As you know, the doctors have talked to (patient's name) about waiting for an organ, but we would like to focus on you to learn from you how to be aware of what family members or caregivers need during this time. Do you have any questions? I would like to record our conversation so that I can focus on what you have to say. Do I have your permission to turn on the recorder?

**TURN ON TAPE NOW.** State study name (THINKER NEXT Study), date and time, interviewer initials and participant ID for recorder.

1. Please tell me about (patient's name) and why doctors think they might need an organ transplant.
2. Please tell me about what doctors have told you about (patient's name) need for an organ transplant.
    a. PROBE: What have doctors told you about the possibility of getting an organ with the hepatitis C virus?
    b. What do you think about that?
    c. PROBE: What have doctors told you about the possibility of getting an organ from someone who has overdosed on drugs?
    d. What do you think about that?
    e. What decision did (patient's name) make about getting an organ that might be from someone with hepatitis C or with a drug overdose?

        i. What role did you play in that decision?

3. (Patient's name) is in the THINKER-NEXT clinical trial that involves getting an organ with the hepatitis C virus.
    a. What do you know about the THINKER-NEXT trial?
    b. What do you know about Hepatitis C?
    c. Where did you get information about getting an organ transplant from someone who has hepatitis C?
        i. What did you think about this information?
        ii. What did you and (patient's name) discuss about this information?
        iii. Please tell me about talking about this information with other family members or friends.
4. What do you think life will be like for YOU after (patient's name) gets a transplant?
    a. How will your life change?
    b. How do you think (patient's name) thinks that life will be like for the two of you after the transplant?
5. How will getting a transplant, especially a transplant with an organ with hepatitis C affect your relationship with other people?
6. What else do you think that doctors need to know about being the family member of a person waiting for an organ transplant?

**TURN OFF TAPE NOW.**

Thank you for talking with me today. Your answers will be very helpful as we try to develop ways to support family members during this process.
