# Supplementary material for: Perspectives of Caregivers of Kidney Transplant Recipients and Transplant Candidates About Kidneys From Donors With Hepatitis C Virus Infection
Source: Kidney Med. 2026 May 13;8(7):101410. doi: 10.1016/j.xkme.2026.101410 (PMC13312101; doi:10.1016/j.xkme.2026.101410)
Supplement: Supplementary File (PDF) — Items S3 [file mmc3.pdf]

S3. Interview guide

Caregivers of people post-transplant

Hello. My name is _______________ and I work with the doctors on the transplant team at Penn/Miami. We are interested in learning about what it is like to be a family member or caregiver of someone who has received a transplant with an organ containing hepatitis C. We want to learn about this so that we can help patients and families during this process. Since you are a family member or caregiver of someone who has received a transplanted organ, we consider <u>you</u> an expert in this. As you know, the doctors have talked to (patient's name) receiving this organ, but we would like to focus on you to learn from you how to be aware of what family members or caregivers need during this time. Everything you say to me will be kept confidential.

While we ask that you let us record this conversation for data collection purposes, the audio will be destroyed once it is transcribed. Any identifiable information, such as your name or where you live, will be removed from the transcription. Anything you share with me will be combined with the responses of other participants before it is shared publicly in manuscripts or presentations.

Before we get started, what questions do you have for me?

TURN ON RECORDER NOW. State study name (THINKER NEXT Study), date and time, interviewer initials and participant ID for recorder.

Please tell me about (patient's name) and why doctors thought they needed an organ transplant.

1. Please tell me about what doctors told <u>you</u> about (patient's name) need for an organ transplant.
    a. How was the actual transplant different from what you expected?
2. (Patient's name) was in the THINKER-NEXT clinical trial that involved getting an organ with the hepatitis C virus.

a. What did you know before the transplant about hepatitis C?
b. What concerns did you have about (patient’s name) getting an organ with hepatitis C?
c. What was it like for you while (patient’s name) was taking the treatment for hepatitis C?
d. What information did you get about hepatitis C?
    i. What did you think about this information?
    ii. What did you and (patient’s name) discuss about this information?
    iii. Please tell me about talking about this information with other family member
e. How do you think the hepatitis C affected <u>you</u>?
f. What did you do differently because (patient’s name) received an organ with hepatitis C?
    i. What do you wish you had known about hepatitis C before the transplant?
    ii. What do you wish you knew now about hepatitis C?
    iii. How did the hepatitis C affected your relationships with other people?

3. What else do you think that doctors need to know about being the family member of a person who gets a transplanted organ with hepatitis C?

Thank you for talking with me today. Your answers will be very helpful as we try to develop ways to support family members during this process.

TURN OFF TAPE NOW.
